# YOU JUST LEARN WHAT TO HOLD ON TO AND WHAT TO LET GO: NARRATIVES OF MINDFUL AGING IN SOUTH ASIAN AMERICAN OLDER ADULTS

**DOI:** 10.1093/geroni/igad104.0830

**Published:** 2023-12-21

**Authors:** Mushira Khan, Ajla Basic

**Affiliations:** Mather Institute, Evanston, Illinois, United States; Mather, Evanston, Illinois, United States

## Abstract

Mindful Sustainable Aging (MSA) is a multidimensional concept incorporating elements from four prominent gerontological theories – activity theory, disengagement theory, successful aging theory, and gerotranscendence theory (Nilsson et al., 2015). Informed by mindfulness practices, MSA is a holistic approach to supporting healthy aging in the social, physical, mental, and spiritual dimensions of life (ibid.). Using MSA as a theoretical framework, this qualitative study explores mindful aging in community-dwelling South Asian American older adults – one of the fastest-growing yet relatively understudied racial/ethnic minority groups in the US. In-depth, semi-structured interviews were conducted with 32 South Asian Americans aged 50+ (18 male, 14 female; mean age=63.5 years). Thematic analysis of the interview data showed that aging was accompanied by significant modifications such as adjusting to everyday life after the death of a spouse, perceived loss of role and status in the family, reduced finances, and limited mobility due to emergent health concerns. Yet, participants expressed an overall sense of peace and gratitude, embracing aging as an “inevitable” and “natural” process of discovery and acquiring wisdom. Participants tried to remain connected to their social networks through messaging and video calling apps like WhatsApp, volunteered at local places of worship like temples and mosques, and helped their adult children with childcare and household chores. Limited availability of culturally-appropriate opportunities for active aging was a key barrier to participants’ sense of well-being. These findings suggest that culturally-sensitive, mindfulness-based interventions like gentle yoga or bilingual guided meditations may enhance subjective well-being in this population sub-group.

